# The METTL3-m^6^A Epitranscriptome: Dynamic Regulator of Epithelial Development, Differentiation, and Cancer

**DOI:** 10.3390/genes12071019

**Published:** 2021-06-30

**Authors:** Alexandra Maldonado López, Brian C. Capell

**Affiliations:** 1 Department of Dermatology, University of Pennsylvania Perelman School of Medicine, Philadelphia, PA 19104, USA; alexmal@pennmedicine.upenn.edu; 2 Department of Genetics, University of Pennsylvania Perelman School of Medicine, Philadelphia, PA 19104, USA; 3 Penn Epigenetics Institute, University of Pennsylvania Perelman School of Medicine, Philadelphia, PA 19104, USA; 4 Abramson Cancer Center, University of Pennsylvania Perelman School of Medicine, Philadelphia, PA 19104, USA

**Keywords:** RNA, epigenetics, epitranscriptomics, m^6^A, METTL3, epithelial, cancer, development, differentiation

## Abstract

Dynamic modifications on RNA, frequently termed both, “RNA epigenetics” and “epitranscriptomics”, offer one of the most exciting emerging areas of gene regulation and biomedicine. Similar to chromatin-based epigenetic mechanisms, writers, readers, and erasers regulate both the presence and interpretation of these modifications, thereby adding further nuance to the control of gene expression. In particular, the most abundant modification on mRNAs, N^6^-methyladenosine (m^6^A), catalyzed by methyltransferase-like 3 (METTL3) has been shown to play a critical role in self-renewing somatic epithelia, fine-tuning the balance between development, differentiation, and cancer, particularly in the case of squamous cell carcinomas (SCCs), which in aggregate, outnumber all other human cancers. Along with the development of targeted inhibitors of epitranscriptomic modulators (e.g., METTL3) now entering clinical trials, the field holds significant promise for treating these abundant cancers. Here, we present the most current summary of this work, while also highlighting the therapeutic potential of these discoveries.

## 1. Introduction to RNA Epigenetics: A New Layer of Gene Regulation

The first RNA modifications were initially described in the 1950s, while N^6^-methyladenosine (m^6^A), the most abundant modification on messenger RNAs (mRNAs), was discovered in the 1970s [1]. In the years, approximately 150 RNA modifications have been described, the majority found on noncoding ribosomal RNA (rRNA) and transfer RNA (tRNA). Considered to be passive structural features for decades, this view was dramatically transformed by the discovery in 2011 that these modifications might in fact be reversible and dynamic. A protein, known as fat mass and obesity-associated protein (FTO) was shown to demethylate m^6^A [2]. These findings, combined with significant advances in the sequencing technologies for profiling these modifications [3], led to the emergence of the terms “RNA epigenetics” and “epitranscriptomics” and an explosion of interest in the field [1,4,5]. 

These diverse RNA modifications now are thought to form an “epitranscriptomic code”, which may enable or enhance RNA-dependent reactions, and change RNA structure-function relationships. Collectively, RNA epigenetics now offers an entirely new layer of regulation to control gene expression in a spatiotemporal manner. As discussed below, given the extensive changes in gene expression, which occur during the self-renewal of epithelial tissues, they provide a great model in which to study the role of epitranscriptomics in both homeostasis and disease states. Here, we summarize the most recent findings at the intersection of RNA epigenetics and epithelial biology. As the most abundant modification on mRNAs, m^6^A has by far been the most studied of the RNA modifications, and thus, will be the major focus of this review. Readers are referred to the following recent excellent reviews for further information on other modifications [5,6,7,8].

## 2. m^6^A: Multifaceted Modulator of mRNAs

m^6^A is conserved across multicellular eukaryotes including plants and vertebrates, single-cell organisms such as archaea, bacteria, and yeast, as well as among viral RNAs. In vertebrates, approximately 25% of mRNAs contain at least one m^6^A modification [9]. In mammals, m^6^A is catalyzed in the nucleus typically at DRACH (D = A/G/U, R = A/G, H = A/C/U) motifs, and is enriched transcriptome-wide in the 3’ untranslated region (UTR), as well as within internal exons and near stop codons [1]. Notably, slower rates of transcription result in increased levels of m^6^A and ultimately reduced efficiency of translation, suggesting that m^6^A deposition relies on the dynamics of the transcribing RNA polymerase II [10]. Consistent with this, the m^6^A catalytic complex can be recruited to DNA by both transcription factors (i.e., CEBPZ) [11], as well as chromatin modifications (i.e., histone H3 lysine 36 trimethylation, or H3K36me3, a modification associated with active transcription) [12]. Collectively, these findings have shown that m^6^A methylation occurs co-transcriptionally. For example, H3K36me3 was shown to be recognized and bound directly by methyltransferase-like 14 (METTL14), a part of the m^6^A catalytic complex, which in turn, binds to an adjacent RNA polymerase II to deposit m^6^A co-transcriptionally [12]. Intriguingly, the interactions between chromatin and RNA modifications appear to move both ways, as m^6^A itself was recently shown to be important for recruiting the histone demethylase, KDM3B, to chromatin in order to demethylate the repressive histone modification, H3K9me2 [13].

As detailed further below, diverse aspects of mRNA metabolism have been shown to have been affected by m^6^A methylation. For example, m^6^A has been shown to either promote mRNA degradation or alternatively its translation by increasing its stability, depending upon the cellular context [1]. Beyond the presence or absence of m^6^A, the amount of m^6^A can also impact the response as highly m^6^A modified transcripts display a slightly lower translational efficiency than unmodified transcripts, suggesting that m^6^A impacts transcript turnover [10]. In addition to affecting mRNA stability, m^6^A may affect mRNA splicing, though evidence in mammalian systems is limited [1,14]. Beyond these direct effects on mRNA stability, a recent study also identified a role for m^6^A methylation on an array of chromosome-associated regulatory RNAs (carRNAs) [15]. Inhibiting m^6^A methylation led to increased chromatin accessibility and nascent transcription in embryonic stem (ES) cells.

Beyond affecting gene expression, some other recent studies have uncovered a novel role for m^6^A in mediating DNA damage and repair processes. For example, it has been shown that m^6^A is deposited upon mRNA rapidly (within ~2 min) at sites of DNA damage following exposure to ultraviolet (UVC) radiation [16], and that in the absence of METTL3 cells showed delayed repair of UVC-induced cyclobutane pyrimidine adducts and increased sensitivity to UVC [16]. Another recent report showed that in response to DNA double-strand breaks (DSBs), METTL3 is activated by ATM-mediated phosphorylation [17]. This phosphorylated METTL3 is then localized to DNA damage sites, where it deposits m^6^A on DNA damage-associated mRNAs, where it ultimately recruits RAD51 and BRCA1 to perform homologous recombination (HR)-mediated repair [17].

Underscoring the potential disease relevance of m^6^A, m^6^A -modified regions are significantly enriched for the presence of single nucleotide polymorphisms (SNPs). Indeed, the number of SNPs decreases as the distance from the m^6^A sites increases [18]. Additionally, another study found that m^6^A -associated quantitative trait loci (QTLs) can significantly alter the binding sites of RNA-binding proteins and have heterogeneous downstream effects, supporting a role for m^6^A in human disease heritability [19]. Together, these emerging studies underscore the incredibly diverse range of biological processes that are impacted by m^6^A, and suggest that much remains to be revealed regarding this critical modification. 

## 3. Writing, Erasing, and Reading the m^6^a Code

### 3.1. The Writers

**Figure 1:**
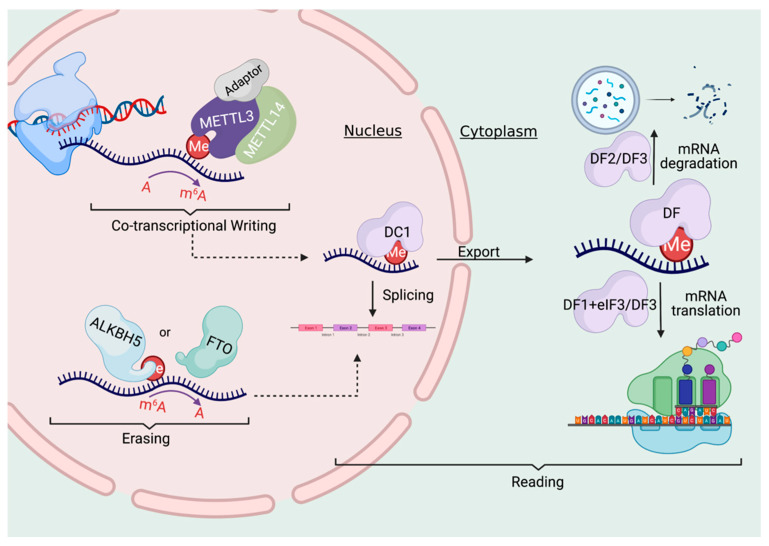
METTL3 catalyzes m^6^A methylation (Me) co-transcriptionally within the nucleus which is facilitated by its interacting partner, METTL14, and a number of adaptor proteins that enhance its activity. A modification upon a mRNA derives in large part by the effects of reader proteins such as the YTH domain-containing RNA binding proteins, and ultimately may lead to mRNA splicing, export, degradation, or translation. For example, while YTHDC1 (DC1) exists in the nucleus to promote nuclear export, YTHDF readers can preferentially promote degradation (i.e., YTHDF2, or “DF2”) or translation (i.e., YTHDF1 and YTHDF3, or “DF1” and DF3” here). Alternatively, m^6^A can also be demethylated by ALKBH5 and FTO in the nucleus.

RNA methylation, similar to the known DNA or histone epigenetic modifications, is regulated by “writers” (methyltransferases), “readers” (RNA binding proteins), and “erasers” (demethylases). m^6^A in mRNA is deposited by a large multicomponent writer complex within the nucleus (Figure 1). METTL3 and METTL14 form a heterodimeric complex that is responsible for the vast majority of m^6^A modifications on Mrna [1,20]. While both METTL3 and METTL14 contain methyltransferase domains, several structural studies demonstrated that only METTL3 contains a binding site for the methylation substrate, S-adenosylmethionine (SAM) [21,22,23]. This demonstrated that METTL3 was the only catalytically active subunit, while METTL14 serves as an allosteric adaptor of METTL3, essential for stabilizing the conformation and substrate RNA binding of METTL3 [24]. METTL3 expression has been shown to correlate with global levels of m^6^A across both human and mouse tissues, supporting its role as the major m^6^A methyltransferase [18]. Beyond METTL3, a very small number of mRNAs have been shown to undergo m^6^A methylation catalyzed by other enzymes. METTL16 carries out m^6^A on U6 small nuclear RNA (snRNA) as well as on methionine adenosyltransferase 2A (MAT2A) [25]. Similarly, another writer called ZCCHC4 methylates A4220 on 28S rRNA [26].

The METTL3/14 heterodimer also binds a number of different adaptors which function in unique ways to facilitate the functions of the complex. For example, Wilms tumor-associated protein (WTAP), though it does not possess methyltransferase activity, binds to METTL3/14 and is required for optimal substrate recruitment and heterodimer localization by binding to chromatin and transcription factors at specific promoters [27,28]. Another adaptor protein, called VIRMA (Vir-like m^6^A methyltransferase associated), is critical for the deposition of m^6^A at 3’ untranslated regions (UTRs) [29]. The ZC3H13 (zinc finger CCCH-type containing 13) adaptor plays a role in the nuclear localization of the complex [30], while RBM15/15B (RNA binding motif protein 15/15B) binds to U-rich regions and can promote the methylation of certain mRNAs [31]. Collectively, these diverse complex members endow the METTL3/14 writer complex with an expanded array of unique abilities to enhance its functions.

### 3.2. The Erasers

While initially, the active demethylation of m^6^A was thought to potentially be an essential part of m^6^A function, more recent findings suggest that it may occur on a limited basis and in a limited number of tissues under physiological conditions. As mentioned above, the first discovered m^6^A eraser was FTO (fat mass and obesity-associated protein) [2], which reportedly removes the methyl group in m^6^A in the nucleus (Figure 1). FTO’s sequence demonstrated some homology to the ALKB family of dioxygenases, which can demethylate both DNA and RNA, providing a clue to FTO possibly having a role in demethylation [32]. However, subsequent studies suggest that FTO may demethylate m^6^A in a very non-specific and inefficient fashion. Indeed, one seminal study demonstrated that FTO displayed much higher catalytic activity for demethylating N6,2′-O-dimethyladenosine (m^6^A_m_), which is found in the 5’UTR [33]. Furthermore, the majority of FTO-mediated demethylation of m^6^A_m_ likely occurs on snRNAs and not mRNAs [34]. In contrast, the other known m^6^A eraser ALKBH5 has no activity towards m^6^A_m_ [33]. ALKBH5 is also localized in the nucleus where it is thought to perform the demethylation reaction [35]. Surprisingly, *Alkbh5*-knockout mice appear generally normal [35], though ALKBH5 is thought to play an important role in spermatogenesis and germ cell development in the testes [35]. Consistent with a potentially more critical role for ALKBH5 in demethylating m^6^A as compared to FTO, expression levels of ALKBH5 are more highly negatively correlated with m^6^A levels in comparison to FTO [18]. As discussed further below, like METTL3 and METTL14, FTO and ALKBH5 are frequently dysregulated in human cancers, suggesting that they may play more critical roles under stress or disease conditions [4].

### 3.3. The Readers

The unique outcomes of m^6^A methylation on mRNA are attributed to the different readers that bind to it. The first discovered m^6^A readers were members of the YTH domain-containing proteins, as the ~150aa YTH domain was demonstrated to bind RNA in a m^6^A-dependent manner. In the mammalian genome, there are five YTH domain-containing proteins, the YTHDCs (YTH domain-containing) and the YTHDFs (YTH domain family) readers. The nuclear m^6^A readers are the YTHDCs. YTHDC1 regulates mRNA splicing by interacting with a variety of splicing regulators such as SRSF3 [36], and also facilitates nuclear export [37] (Figure 1). Notably, YTHDC1 has been reported to preferentially bind ncRNAs rather than mRNAs [31]. YTHDC2, which is highly expressed in the testes, has been shown to both promote mRNA degradation, as well as translation, suggesting this reader may perform context- and tissue-specific functions [1]. 

YTHDF proteins bind m^6^A in the cytoplasm (Figure 1), and in turn, can affect mRNA stability, translation, and/or localization. YTHDF1 has been proposed to bind to eIF3 to promote translation [38], while YTHDF2 contributes to the mRNA destabilizing effect of m^6^A and promotes the degradation of the mRNA [39]. YTHDF3 has been shown to promote both the degradation and translation of m^6^A-marked mRNAs [40]. Despite this, emerging evidence suggests that all of YTHDF proteins may primarily serve to promote mRNA degradation [41,42]. Mechanistically, this occurs when the YTHDF-bound, m^6^A modified mRNAs undergo phase separation into liquid droplets such as stress granules and P-bodies that are then processed [9]. Beyond, YTH domain-containing proteins, the IGF2BP (IGF2BP1, 2, 3) proteins, as well as fragile X mental retardation protein (FMRP), weakly bind m^6^A, but when bound, can promote its stability and translation. Together, these emerging findings highlight both the nuance and complexity of RNA epigenetic regulation and how it may contribute to cellular phenotypes, as well as the need for further study to clarify these distinct findings.

### 3.4. Methods for Studying m^6^A

Fundamental to all the recent advances in the epitranscriptomics field has been the discovery of new methods to detect and study these modifications genome-wide. The earliest and most widespread of these for m^6^A were the antibody-based approaches known as m^6^A immunoprecipitation-sequencing (m^6^A-seq), as well as methylated RNA immunoprecipitation sequencing (MeRIP-seq) [43]. However, antibody approaches have some limitations. First, there is the issue of non-specificity. For example, m^6^A-seq is unable to differentiate between m^6^A and N6,2′-O-dimethyladenosine (m^6^Am). Second the resolution of these approaches is in the hundreds of base pairs. These limitations have driven the development of methods that are antibody-free and limited to a single nucleotide resolution. 

For single nucleotide resolution studies, significant improvement is obtained by adding a UV crosslinking step after the formation of non-covalent complexes between m^6^A-modified transcripts and the m^6^A antibody to induce mutations during reverse transcription. The more precise localization of m^6^A peaks called miCLIP or m^6^A-CLIP [43]. It has the disadvantage of requiring much more starting material than the m^6^A-seq or the MeRIP-seq. 

Antibody free approaches include DART-seq (deamination adjacent to RNA modification targets), which uses APOBEC1 (a cytidine deaminase) fused to the YTH domain of YTHDF2 [44]. This fusion induces C-to-U deamination at sites adjacent to m^6^A residues that are then detected using RNA-seq, using as little material as 10 ng of total RNA. Endoribonuclease-based approaches include the MAZTER-seq method which relies on the ability of the bacterial RNase MazF to cleave RNA upstream of an “ACA” sequence, but it does not cleave upstream of “m^6^A-CA” and can map m6A at single nucleotide resolution [45]. In addition, m^6^A-sensitive RNA-endoribonuclease–facilitated sequencing (m^6^A-REF-seq) uses the m^6^A-sensitive endoribonuclease ChpBK to identify and quantify transcriptomic m^6^A sites at specific motifs at single base resolution [46].

Finally, complementing these sequencing-based methodologies are biophysical methods, such as thin layer chromatography and mass spectrometry that can serve as powerful tools for both validation and the identification and quantification of m^6^A [43].

## 4. Self-Renewing Epithelia: The Need for Dynamic Gene Regulatory Control

Dynamic, self-renewing tissues like epithelia undergo dramatic alterations in gene expression as they differentiate, suggesting that RNA epigenetic mechanisms may play important roles in their physiology. Epithelia are found lining internal and external surfaces, such as the lining of the intestinal track and the surface of the skin, and form the major barriers between the internal and external environments. The different types of epithelia are characterized as being stratified, transitional, or glandular, and depending on the region of the body, the cells can vary in shape and amount of stratification [47]. These tissues can be present in either dry or wet environments, such as the epidermis, and the oral cavity, respectively. The stratified non-cornified epithelia is found in the epithelium of the oral cavity, esophagus, vagina and urethra. Stratified-cornified epithelia have epithelial cells that are cornified and dead, thus called corneocytes, and are found in the epidermis and at the plate of the human fingernail [48]. 

In stratified epithelia, basal stem-like progenitor cells adhere to the basement membrane and through a continuous regenerative process, go on to replace the “suprabasal” cells above them [49]. With further differentiation, the surface epithelial cell eventually slough off and the process continues [50,51]. This dynamic self-renewal process depends on the presence of the keratinocyte stem cells and their ability to undergo profound physiological and transcriptional changes. For example, keratin genes, which produce the namesake intermediate filaments of keratinocytes that account for about 80% of the total protein content in differentiated cells of stratified epithelia, display unique expression patterns that change with the state of differentiation. Keratin 14 (K14) is highly expressed in the basal cells of stratified epithelia and is correlated with proliferative activity of keratinocytes. In contrast, Keratin 10 (K10) is highly expressed in the post-proliferative cells of the suprabasal epidermis [48,52]. 

Any dysregulation of this highly coordinated differentiation process can lead to hyperplasia, abnormal differentiation, and diseases ranging from the neoplastic (squamous cell carcinoma) to the inflammatory (i.e., psoriasis). Indeed, while significant progress has been made in unraveling many of the pathways and players involved in driving these processes across epithelial tissues, such as WNT, Notch, Sonic Hedgehog (Shh), and BMP signaling [49,50,51], as well as the master epithelial transcription factor p63 [47,53], the role for epigenetic regulation, and particularly that of RNA epigenetics, is poorly understood. 

## 5. RNA Epigenetics in Epithelial Development and Differentiation

As suggested by the ability of METTL3-mediated m^6^A to promote either mRNA degradation or translation, perhaps it is not surprising that altering METTL3-m^6^A may lead disparate outcomes on differentiation depending upon the timing of *Mettl3* deletion during development. Notably, a complete absence of m^6^A due to *Mettl3* deletion is early embryonic lethal. Knockout of METTL3 from both human and mouse ES cells results in increased transcript half-life of key pluripotency regulators such *Nanog*, *Sox2*, and *Klf4*, improves self-renewal and proliferative abilities, and prevents differentiation from naïve pluripotency into downstream lineages [54,55]. In contrast, and as discussed further in the next section, in cancer, METTL3-mediated m^6^A is thought to impair differentiation and promote carcinogenesis in a variety of cancer contexts [56], such as acute myeloid leukemia [57] and cutaneous squamous cell carcinoma [58]. 

In terms of our understanding of m^6^A’s roles during epithelial homeostasis and differentiation, studies are very limited. One recent examination utilizing a Krt14-Cre recombinase to delete *Mettl3* in epidermis and oral epithelium demonstrated broad developmental defects including a significant failure of hair morphogenesis, premature interfollicular differentiation, and a loss of filiform papillae in the tongue. The authors attributed these effects to altered Wnt signaling [59]. Another study demonstrated that deletion of *Mettl14* in the murine epidermis impaired the m^6^A-dependent association between the long non-coding RNA (lncRNA), *Pvt1*, and MYC. This interaction was shown to be critical for the promotion of epidermal stemness and wound healing capabilities [60]. These significant phenotypic abnormalities demonstrate both, the critical role for m^6^A in epithelial homeostasis, as well as the need for future studies to answer all of the outstanding questions at the intersection of the epitranscriptome and normal epithelial functions.

## 6. The METTL3-m^6^A Epitranscriptome: Key Player in Epithelial Cancers

**Figure 2:**
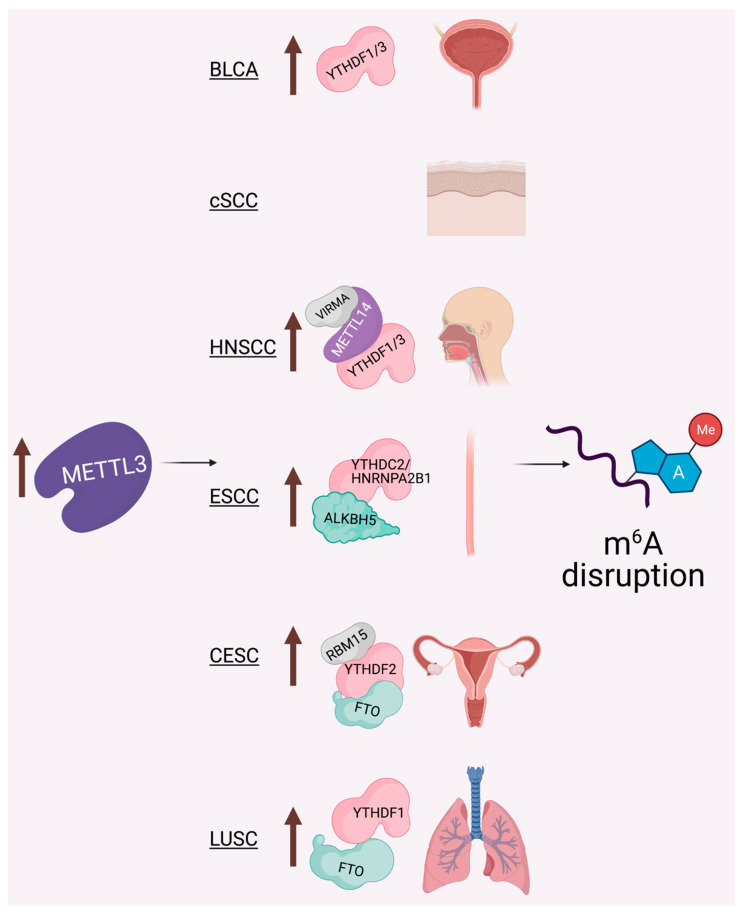
Epithelial cancers, and SCCs in particular, share numerous common biological underpinnings, including dysregulation of the METTL3-m^6^A epitranscriptome. To date, studies have demonstrated consistent overexpression of METTL3 across these cancers. As detailed further in the text and Table 1, depending upon the cancer, other writers (purple), readers (pink), erasers (green), and adaptors (gray) have been shown to display dysregulated expression or activity, ultimately driving m^6^A disruption and carcinogenesis through diverse mechanisms, such as oncogene activation or therapy resistance. Cancers listed include bladder cancer (BLCA), cutaneous SCC (cSCC), head and neck SCC (HNSCC), esophageal SCC (ESCC), cervical SCC (CESC), and lung SCC (LUSC).

Regarding disease contexts, no area has been better studied than cancer in the RNA epigenetics field [4,61,62]. As we highlight further below, METTL3 and m^6^A in particular have been implicated in numerous aspects of epithelial cancer biology. Similar to the way in which SCCs share numerous common biological underpinnings [63,64], the disruption of METTL3 and m^6^A function can promote similar broad effects across the diverse forms of SCC (Figure 2 and Table 1).

### 6.1. Cutaneous Squamous Cell Carcinoma (cSCC)

cSCC, originating in the epidermis, is the second most common of all human cancers and its incidence is increasing with the aging of the population. While typically treatable with surgery, up to 5000–8000 people die every year in the United States (U.S.) secondary to metastatic cSCC [65], numbers rivaling that of melanoma. Recently, it was demonstrated that METTL3 is frequently overexpressed in cSCC patient samples. Upon METTL3 knockdown in vitro, this same study reported impaired cellular proliferation and self-renewal along with the promotion of cSCC cell differentiation through the reduction of p63 and K14 (markers of basal stem cells), in combination with enhanced expression of K10 (a marker of keratinocyte differentiation) [58]. When considered with the previously discussed results demonstrating the critical role of METTL3-mediated m^6^A in responding to UV-induced DNA damage [16], the major driver of cSCC, these studies highlight not just the importance but also the complexity that the epitranscriptome might play in the initiation and progression of cSCC. Future in vivo studies will likely help further elucidate the mechanisms behind these observations and offer insights into other epitranscriptomic regulators as they pertain to this pervasive cancer. 

### 6.2. Head and Neck Squamous Cell Carcinoma (HNSCC) 

Head and neck squamous cell carcinoma (HNSCC) is the sixth most common of all human cancers worldwide, and despite advances in cancer therapies, it continues to have a relatively poor prognosis and high rate of morbidity and mortality [66]. HNSCC consists of malignant tumors that occur on the mucosal surfaces of the upper respiratory digestive tract which involves the nasal cavity, paranasal sinuses, nasopharynx, hypopharynx, larynx, trachea, oral cavity, and oropharynx. A variety of studies have been performed to examine RNA epigenetic regulators which we briefly summarize here. 

Seeking to identify potential biomarkers in HNSCC, one recent study examined data from The Cancer Genome Atlas (TCGA) to explore relationships between expression levels of m^6^A regulatory genes and the survival rate of HNSCC patients [67]. This study identified a broad upregulation of these genes in tumor samples as compared to normal tissues, with significant increases in METTL3, METTL14, KIAA1429 (VIRMA), YTHDF1, YTHDF2, ALKBH5, FTO, WTAP, RBM15, and HNRNPC. In contrast, YTHDC2 was significantly downregulated. In terms of predicting prognosis, patients with higher YTHDC2 or lower HNRNPC expression showed greater overall survival [67]. Another study performed a similar analysis and found that VIRMA was the most frequently altered m^6^A regulatory gene, followed by YTHDF3, METTL3 and YTHDF1. The increased expression of VIRMA was associated with higher cancer stages, tumor grade, and nodal metastasis, suggesting that its dysfunction may play an important role in the initiation and progression of HNSCC [68]. Another group also explored TCGA data to focus on how the expression of lncRNAs may impact HNSCC tumorigenesis. They identify the lncRNA LNCAROD to play a potentially oncogenic role in HNSCC [69]. Mechanistically, they found that METTL3/14-mediated m^6^A enhanced the stability of LNCAROD, which then protected the oncogenic protein YBX1 from proteasomal degradation to promote HNSCC cell proliferation and mobility in vitro and tumorigenic ability in vivo [69]. Additionally, in the nasopharyngeal form of HNSCC, m^6^A was shown to reduce the expression of ZNF750, an important pro-differentiation tumor suppressor [70].

Oral squamous cell carcinoma (OSCC), a form of HNSCC, is the major subtype of oral cancer, responsible for approximately 90% of oral neoplasms [66]. It is characterized by a high rate of recurrence and metastasis, as well as a poor response to clinical therapies. Various studies have shown that METTL3 is significantly upregulated in human OSCC tissues and cells. One of these studies showed that METTL3 mediates the stabilization of the MYC mRNA by 3’UTR m^6^A methylation in a YTHDF1-dependent manner [71]. Depleting METTL3 led to reduced c-Myc protein levels and reduced proliferation and migration of OSCC cells in vitro and tumorigenicity in vivo [71]. Another study found similar results upon METTL3 knockdown, but attributed their findings to a different mechanism. Here they observed that METTL3-mediated m^6^A methylation of BMI1 promoted its translation in conjunction with IGF2BP1 [72]. They showed that inducible deletion of *Mettl3* in vivo inhibited the development of OSCC upon carcinogen exposure [72]. Future studies will likely clarify the underlying mechanisms, but the abundance of evidence has now shown that METTL3-mediated m^6^A can clearly play an oncogenic role in OSCC pathogenesis, and suggests that targeting METTL3 pharmacologically may be a therapeutic approach worth testing. 

### 6.3. Cervical Squamous Cell Carcinoma (CESC)

CESC is the fourth most diagnosed cancer and the fourth leading cause of cancer-associated mortality in women worldwide [73]. Two groups recently utilized TCGA data and identified that METTL3, YTHDF2 and RBM15 were significantly increased in CESC, while the expression of FTO was significantly decreased [74,75]. In contrast, other studies have found FTO to be overexpressed in CESC. One report demonstrated that inhibition of FTO impaired the proliferation and migration of CESC cell lines [76]. Mechanistically, they suggested this occurred through impaired translation of both MYC and E2F1 [76]. Similarly, another study found that FTO overexpression increased chemoradiotherapy resistance of CESC [77]. FTO was shown to be overexpressed in human CESC tissues and increased FTO levels correlated with poor survival. This was driven by FTO’s ability to reduce m^6^A and subsequently increase the expression levels of β-catenin mRNA and protein in CESC cells. The increased β-catenin resulted in increased levels of the nucleotide excision repair factor (ERCC1) in CESC. By knocking down β-catenin, ERCC1 levels were reduced, and the cells (and particularly those with FTO overexpression) were sensitized to chemoradiotherapy [77]. 

### 6.4. Lung Squamous Cell Carcinoma (LUSC)

Lung cancer is the principal cause of cancer-related death with an approximate 5-year survival rate of 16.6% [78]. There are 2 main types of lung cancer: non-small-cell lung cancer (NSCLC) and small-cell lung cancer (SCLC). NSCLCs account for 80% of lung cancers and within them there is two major subtypes which are lung adenocarcinoma (LUAD) and lung squamous cell carcinoma (LUSC). These two are responsible for 50–60%, and 30% of lung cancer cases, respectively. A recent study analyzed the correlation between m^6^A regulatory gene expression and patient prognosis utilizing TCGA data. This analysis revealed that higher FTO expression was associated with a poor prognosis in LUSC patients (Figure 2). FTO knockdown led to decreased m^6^A levels, reduced proliferation and invasiveness, and increased apoptosis in cell lines. In relation to the mechanism of these findings, the authors found that FTO significantly increased MZF1 expression in LUSC by reducing its m^6^A levels and in turn increasing its mRNA stability and translation [79]. Another study identified that the reader YTHDF1 is amplified in various types of cancers including NSCLC. They show that knockdown of YTHDF1 dramatically stunted tumor formation, tumor weights, and volumes in a NSCLC xenograft model [80]. Surprisingly, when looking at human patient data that observed that high YTHDF1 expression was associated with improved clinical outcomes, as reduced YTHDF1 impaired responses the chemotherapy [80]. Finally, in lung adenocarcinoma, METTL3 has been reported to be upregulated and play an oncogenic role in promoting the growth, survival, and invasion of human lung cancer cells by promoting the translation of certain mRNAs, such as EGFR, TAZ, and BRD4 [38,81]. 

### 6.5. Bladder Cancer (BLCA) 

Urothelial carcinoma of the bladder is currently the fourth most common malignancy in men [82]. A recent study found that METTL3 is significantly upregulated in BLCA tissues and that patients with high expression of METTL3 had a worse prognosis and shorter overall survival compared to those with low expression of METTL3 (Figure 2) [83]. The authors found that METTL3 could bind to microprocessor protein DGCR8 to promote the increased expression of micro-RNAs (miRNAs), which collectively played an oncogenic role in BLCA [83]. In a similar fashion, another group also observed the ability of METTL3-mediated m^6^A to promote the expression of an oncogene (CDCP1) that together could promote malignant transformation and the progression of BLCA in vitro and in vivo [84]. Another study identified that the adhesion molecule integrin alpha-6 (ITGA6) undergoes dynamic m^6^A regulation by METTL3 and ALKBH5. Their results revealed that depletion of METTL3 or overexpression of ALKBH5 in BLCA cells resulted in reduced ITGA6 translation and decreased cell adhesion in a m^6^A-dependent manner [85]. Their data suggests that METTL3-mediated m^6^A in the 3′UTR of ITGA6 promotes its translation in conjunction with YTHDF1 and YTHDF3, and collectively this promoted the growth and metastatic capacity of BLCA cells [85]. Similarly, another group also showed that METTL3 mRNA expression is significantly elevated in BLCA, while expression of METTL14, WTAP, FTO and ALKBH5 were not significantly different in the same patient samples [86]. This group showed that METTL3 overexpression promotes BLCA cell proliferation and migration, while suppressing apoptosis in vitro by catalyzing m^6^A on MYC, AFF4, RELA and IKBKB to promote their activation [86].

**Table 1:** Summary of mechanistic studies into the role of the METTL3-m^6^A epitranscriptome in epithelial cancers, and particular SCCs.

SCC	Mechanism(s)	Reference(s)
cSCC	Increased METTL3-m^6^A promotes upregulation of p63 and K14, downregulation of K10, and cell proliferation	[58] Zhou, R. et al., 2019
HNSCC	METTL3 adds m^6^A to cMyc mRNA to enhance its stability and promote proliferation, invasion and migration in vitro and tumorigenicity in vivo	[71] Zhao, W. et al., 2020
	METTL3 adds m^6^A to BMI1 mRNA to promote its translation in conjunction with IGF2BP1 to drive tumorigenesis	[72] Liu, L. et al., 2020
	Increased METTL3/14-m^6^A enhances LNCAROD which protects the oncogenic protein YBX1 from degradation to drive tumorigenesis	[68] Arumugam, P. et al., 2021
CESC	FTO is frequently overexpressed and removes m^6^A to lead to the increased translation of MYC, E2F1, and b-catenin, promoting both radiotherapy resistance and poor survival	[76] Zou, D. et al., 2019; [77] Zhou, S. et al., 2018
LUSC	High YTHDF1 expression promotes tumorigenesis in murine models, though in humans increased expression associated with better responses to chemotherapy and improved clinical outcomes	[80] Shi, Y. et al., 2019
	FTO overexpression removes m^6^A to lead to the increased translation of MZF1 which promotes proliferation and invasion	[79] Liu, J. et al., 2018
BLCA	Increased METTL3-m^6^A in conjunction with YTHDF1 and YTHDF3 promotes ITGA6 mRNA translation which enhances the growth and metastasis of BLCA cells	[85] Jin, H. et al., 2019
	Increased METTL3 associated with worse prognosis and survival, promotes increased miRNA and CDCP1 oncogene expression to enhance malignant transformation in vitro and in vivo	[83] Han, J. et al., 2019; [84] Yang, F. et al., 2019
ESCC	Increased METTL3 associated with worse survival and promotes proliferation via increasing AKT expression	[87] Hou, H. et al., 2020
	YTHDC2 variants associated with ESCC and its inhibition blocks ESCC cell proliferation	[88] Yang, N. et al., 2020
	Increased reader expression (HNRNPA2B1) correlates with tumor diameter and lymphatic metastasis and promotes disease and its knockdown can block proliferation, migration, and invasion	[89] Guo, H. et al., 2020

While there are some underlying commonalities, the findings give a sampling of the diverse mechanisms by which dysregulation of the writers, readers, and erasers of m^6^A can promote cancer in a context- and tissue-specific fashion. Cancers listed include bladder cancer (BLCA), cutaneous SCC (cSCC), head and neck SCC (HNSCC), esophageal SCC (ESCC), cervical SCC (CESC), and lung SCC (LUSC).

### 6.6. Esophageal Squamous Cell Carcinoma (ESCC) 

Esophageal carcinoma is one of the most common malignant tumors worldwide, in which 90% of these are esophageal squamous cell carcinomas (ESCC) [90]. Despite the advances in treatment, the prognosis of patients with ESCC is still poor with the 5-year overall survival rate ranging from 20% to 30%. METTL3 has been shown to be upregulated in ESCC tissues and correlate with worse prognosis [91,92,93]. The inhibition of METTL3 inhibited the proliferation in esophageal cancer cell lines via inhibition of AKT signaling [87]. One recent study found that variants in the *YTHDC2* gene were associated with ESCC risk, and that knocking down YTHDC2 impaired the proliferation rate of ESCC cells [88]. Another study reported that 14 out of 19 assessed m^6^A regulatory genes displayed increased expression in esophageal cancer tissue in comparison to normal tissue [89]. In particular, they found that high expression of ALKBH5 and, in particular, another m^6^A reader, HNRNPA2B1, correlated with tumor diameter and lymphatic metastasis and promoted disease by upregulating fatty acid synthesis enzymes, ACLY and ACC1. Consistent with this, knockdown of HNRNPA2B1 could block proliferation, migration, and invasion in esophageal cancer models [89]. 

In summary, these findings demonstrate the numerous and frequently context-dependent manner by which the dynamic METTL3- m^6^A epitranscriptome may contribute to carcinogenesis. In addition to all of the above examples from specific epithelial cancers, many other studies have highlighted other broad mechanisms that m^6^A may affect to promote cancer. For example, m^6^A has been shown to directly impact interferon signaling by leading to the decay of interferon gene mRNAs [94,95]. As a key regulator of antitumor immunity, as well as responses to immunotherapies, this may indeed be a major additional mechanism through which m^6^A could promote tumorigenesis. Additionally, METTL3- m^6^A has been shown to promote epithelial to mesenchymal transition (EMT) in several studies [96,97,98,99], an important driver of metastasis in epithelial cancers. Collectively, these emerging data again highlight the complexity of m^6^A, as well as the need to further understand all of the underlying mechanisms of its roles in cancer, to improve therapeutic outcomes. 

## 7. RNA Modifications Beyond m^6^A and Roles in Carcinogenesis

As alluded to previously, there are numerous RNA modifications beyond m^6^A, many of which have been implicated in the balance between differentiation and cancer [6,61,100]. For example, 5-methylcytosine (m^5^C) has been detected not only on rRNAs and tRNAs, but also on mRNAs by transcriptome-wide mapping approaches. This m^5^C on mRNAs has been shown to be catalyzed by NOP2/Sun RNA methyltransferase family member 2 (NSUN2). NSUN2 is overexpressed in a variety of human cancers, including cSCC, colon, and breast cancers, and its inhibition impaired MYC-dependent proliferation in keratinocytes and cSCC xenograft studies [101]. In cSCC models, NSUN2 is a critical mediator of chemotherapy resistance [102]. A recent study in BLCA showed that numerous oncogenes are hypermethylated with m^5^C promoting their translation and the invasiveness and metastasis of BLCA. The authors found that NSUN2 and the m^5^C reader YBX1 promoted the expression of the oncogene, HDGF, in a m^5^C dependent manner, and that higher expression of this NSUN2-YBX1-HDGF axis correlated with poor prognosis [103]. NSUN2 was recently shown to bind to and increase the expression of the lncRNA, NMR, in ESCC. NMR was further demonstrated to be significantly upregulated in 119 ESCC tumor tissues and its increased expression correlated significantly with poor overall survival and tumor stage in ESCC patients. The knockdown of NMR significantly reduced ESCC cell migration and invasion showing that it has an oncogenic effect in ESCC cells [104].

## 8. Conclusions and Future Perspectives

More than 150 RNA post-transcriptional modifications have been identified to date, which are widely distributed on various types of RNA, including mRNA, tRNA, rRNA, small non-coding RNA (sncRNA), and lncRNA. The variety and breadth of these numerous modifications and numerous types of RNA underscores the vast potential they have to regulate biology and disease. On this basis, RNA epigenetic (epitranscriptomic) modifications have now been shown to play important roles in numerous critical physiological aspects of tissue development and disease. Despite this, there are many unknowns in this relatively nascent field. While the initial insights into the role of the epitranscriptome on epithelial biology and disease have been exciting, much remains to be discovered.

RNA epigenetics provides for an entirely new layer of gene regulation, offering impressive nuance and complexity to the regulation of dynamic tissues, such as self-renewing epithelia. Indeed, given the ability to target the enzymes that write and erase these modifications pharmacologically, there is significant promise for the promise of epitranscriptome-targeting drugs to treat disease, as attested to by the significant number of companies that have sprung up to develop these compounds. However, as our summary above suggests, a great deal remains to be done, in order to better understand the mechanisms through which RNA modifiers impact epithelial disease first, so until then it is clear that the RNA epigenetics/epitranscriptomics field will remain one of the most exciting areas of biomedical discovery for years to come.
